# Chronic morphine exposure potentiates p-glycoprotein trafficking from nuclear reservoirs in cortical rat brain microvessels

**DOI:** 10.1371/journal.pone.0192340

**Published:** 2018-02-07

**Authors:** Charles P. Schaefer, Nathan B. Arkwright, Leigh M. Jacobs, Chelsea K. Jarvis, Kristen C. Hunn, Tally M. Largent-Milnes, Margaret E. Tome, Thomas P. Davis

**Affiliations:** Department of Pharmacology, University of Arizona, Tucson, Arizona, United States of America; Temple University, UNITED STATES

## Abstract

The rates of opioid prescription and use have continued to increase over the last few decades resulting in a greater number of opioid tolerant patients. Treatment of acute pain from surgery and injury is a clinical challenge for these patients. Several pain management strategies including prescribing increased opioids are used clinically with limited success; all currently available strategies have significant limitations. Many opioids are a substrate for p-glycoprotein (p-gp), an efflux transporter at the blood-brain barrier (BBB). Increased p-gp is associated with a decreased central nervous system uptake and analgesic efficacy of morphine. Our laboratory previously found that acute peripheral inflammatory pain (PIP) induces p-gp trafficking from the nucleus to the luminal surface of endothelial cells making up the BBB concomitant with increased p-gp activity and decreased morphine analgesic efficacy. In the current study, we tested whether PIP-induced p-gp trafficking could contribute to decreased opioid efficacy in morphine tolerant rats. A 6-day continuous dosing of morphine from osmotic minipumps was used to establish morphine tolerance in female rats. PIP induced p-gp trafficking away from nuclear stores showed a 2-fold increase in morphine tolerant rats. This observation suggests that p-gp trafficking contributes to the decreased morphine analgesic effects in morphine tolerant rats experiencing an acute pain stimulus. Attenuating p-gp trafficking during an acute pain stimulus could improve pain management by increasing the amount of opioid that could reach CNS analgesic targets and decrease the need for the dose escalation that is a serious challenge in pain management.

## Introduction

Long-term opioid use, even in a therapeutic setting, will lead to the development of opioid analgesic tolerance [1]. Opioid tolerance is a state in which a patient requires increasing doses of opioids to achieve the same analgesic effect. Addressing the problem of acute pain management for opioid-tolerant patients is an increasingly pressing clinical challenge. Improper pain management is costly to both patients and caregivers. Increased doses of opioids is one strategy used to overcome the issue of tolerance and manage acute pain, however, this approach has not worked well and has been partly responsible for our current opioid epidemic. Other strategies are also used including Non-Steroidal Anti-Inflammatory Drugs and local anesthetics to manage acute pain in these patients [2]. Treatment strategies involving NMDA receptor antagonists as a fast acting therapy for pain management in opioid tolerant patients have been attempted [3]. Ketamine is the drug that has been used clinically, but the interaction between ketamine and opioids is complex and not fully understood, leading to further complications [3–6]. Surgical patients can benefit from complex plans consisting of pre-operative opioid dose tapering with an increase in opioid dose after the procedure [2]. This strategy is only an option for planned procedures and cannot be used in an emergency, giving this strategy limited clinical application. All of these methods to treat pain in opioid-tolerant patients have significant limitations. There is a critical need for a better understanding of the biology and pharmacology underlying opioid tolerance and concurrent pain responses to devise more effective pain management strategies.

Central to opioid analgesia and tolerance are the effects on the central nervous system (CNS). Opioid access to the CNS is regulated in large part by the ability of opioids to cross the blood brain barrier (BBB) into the CNS. The BBB is both a physical and biochemical barrier primarily caused by the characteristics of the endothelial cells that line the lumen of brain capillaries [7]. The tight junctions between endothelial cells creates a physical barrier that limits paracellular diffusion [7,8]. Transcellular movement of compounds is regulated by a biochemical barrier. The biochemical barrier, created by a series of efflux transporters-particularly the ABC transporters, is responsible for many of the therapeutic challenges associated with the BBB. P-glycoprotein (p-gp) (MDR1/ABCB1, E.C. 3.6.3.44) is the most abundant efflux transporter in the human BBB. P-gp, an ABC transporter, utilizes ATP hydrolysis to transport substrates against their concentration gradient [9]. This action allows p-gp to transport an impressive array of blood-borne substrates ranging from xenobiotics to therapeutic drugs back into circulation [10]. The prototypical clinically used opioid, morphine, is a known substrate of p-gp. Increased p-gp at the capillary lumen is associated with decreased morphine efficacy [11,12].

Regulation of p-gp is complex (recently reviewed in [10,13]). Transcriptional and translational regulatory mechanisms control protein amounts; protein trafficking and activation mechanisms contribute to acute regulation of activity and the ability to efflux drugs into the capillary lumen. Opioid tolerance is associated with an increase in p-gp expression in specific brain regions and an increase in basal p-gp activity that is dependent on the presence of substrate [14–16]. Whether morphine tolerance impacts acute regulation of p-gp is unknown.

Using a model of peripheral inflammatory pain (PIP) induced by an injection of λ-carrageenan into a rat’s hind paw we previously examined acute regulation of BBB integrity. In rats, PIP induces changes in the BBB by altering components of both paracellular and transcellular transport. The acute phase of the PIP response (less than 3h post-stimulus) is characterized by increased paracellular leak as measured by an increase in the ability of sucrose to cross the BBB [17]. Increased leak is accompanied by alterations in the tight junction protein oligomeric structures and cellular location [18]. The acute phase of the PIP response is also characterized by decreased morphine uptake into the brain and decreased analgesic efficacy [12]. This effect can be reversed by administration of a nerve block, demonstrating a clear relationship between nociceptive relay and reduction of the analgesic effect of morphine [19]. The decrease in morphine analgesic efficacy is accompanied by redistribution of p-gp within membrane microdomains [20] and trafficking of p-gp from nuclear reservoirs to the luminal surface of the brain endothelial cells [21]. This trafficking moves p-gp to an intracellular location where it can function to increase the export of substrates, including opioids, into the blood. Measurements at timepoints greater than 3h from the stimulus indicate changes that are more characteristic of altered transcription and translation [10,13,17].

We hypothesized that p-gp trafficking could contribute to decreased opioid analgesic efficacy during acute pain in opioid tolerant animals. In this study, we used osmotic minipumps to model long-term opioid exposure in female rats. We sampled cortical microvessels 3h after λ-carrageenan injection to measure the acute p-gp trafficking response to the stimulus. We found that morphine exposure was sufficient to cause a 2-fold increase in p-gp trafficking away from the nucleus in cortical microvessels when animals were exposed to PIP.

## Materials and methods

### Reagents

Morphine was acquired from the National Institute on Drug Abuse (Bethesda, MD). Tris (2- carboxyethyl) phosphine hydrochloride, 4x sample loading buffer and Precision Plus prestained molecular weight standards were purchased from Bio-Rad (Hercules, CA). All other chemicals were acquired through Sigma-Aldrich (St. Louis, MO) unless otherwise stated.

### Animals and treatments

All animal protocols used in these studies were in compliance with the guidelines of the National Institutes of Health and the International Association for the Study of Pain and approved by the University of Arizona Institutional Animal Care and Use Committee (Protocol # 09–029). Results were reported according to the ARRIVE guidelines. Female Sprague-Dawley rats (175–200 g; Envigo, Indianapolis, IN) were cared for using the standard conditions in the University of Arizona Animal Care Facility. All animals were allowed to acclimate for one week before being used in any experiment. Animals were randomly assigned to experimental groups.

### Induction of peripheral inflammatory pain

A 0.1 mL injection of either λ-carrageenan (3% in 0.9% saline) or 0.9% saline was injected into the left hind paw of animals 3 hours before behavioral measurements and sacrifice.

### Pump insertion surgery

Alzet (Cupertino, CA) osmotic minipumps were filled to maximum capacity with morphine sulfate dissolved in 0.9% saline or 0.9% saline. Morphine concentration was calculated so that the pump delivered 5 mg/kg/day to a rat weighing 200 g. Pumps were primed by incubation in 0.9% saline overnight at 37°C. For subscapular pump insertion, rats were anesthetized under 5.0% isoflurane in air and maintained at 2.5% isoflurane in air during surgery. Minipumps were weighed empty, after filling, after priming, and after removal to monitor proper function. Remaining volume in the minipump was also determined at sacrifice. All pumps were implanted 6-days prior to λ-carrageenan injection.

### von Frey mechanical sensitivity

Two people were present for all behavior studies. The assessment of mechanical hypersensitivity was determined using the up-down method described by Dixon *et al*. [22]. Briefly, the rats were placed into the chambers for at least 10 minutes to allow them to acclimate before any measurements were taken. Calibrated filaments were applied perpendicularly to the plantar surface of the hind paw while in individual Plexiglas chambers suspended on a wire-mesh. Rats were treated with an acute dose (2.5 mg/kg) of morphine in 0.9% saline or 0.9% saline 3 hours after injection of 0.1 mL λ-carrageenan or saline into the left hind paw. Mechanical sensitivity was measured before surgery, before λ-carrageenan injection, before morphine injection, and at 10, 20, 30, 45, 60, 90, 120, and 150 minutes after acute injection of morphine. Pre-surgery and pre-λ-carrageenan injection values were used to determine opioid-induced allodynia. Animals that achieved a maximal threshold score (15 g) following exposure were excluded from these calculations. The 50% withdrawal threshold was determined by using the algorithm in Chaplan et al. [23]. Area under the curve (AUC) was determined by calculating the sum of the area under each set of consecutive points starting at the first elevated point and ending at the last elevated point.

### Hargreaves’ thermal sensitivity

Thermal sensitivity was tested using the method described by Hargreaves *et al*. [24]. The infrared emitter was calibrated to deliver 190 mW/cm^2^, as per the manufacturer’s calibration instructions. Briefly, rats were placed into the chambers for at least 10 minutes to allow them to acclimate before any measurements were taken. The rats were treated similarly to those in the von Frey test of mechanical sensitivity. The infrared emitter was placed under each foot and turned on. Time to paw withdrawal (seconds) was measured using a laboratory timer and was started and stopped by the person operating the infrared emitter. Thermal sensitivity was measured before surgery, before λ-carrageenan injection, before morphine injection, and at 10, 20, 30, 45, 60, 90, 120, and 150 minutes after injection of morphine. AUC was calculated similar to the von Frey data.

### Paw edema

Paw edema was measured 3 hours after λ-carrageenan (or saline) injection, 30 minutes after morphine injection, and 150 minutes after morphine injection. A Ugo-Basile (Varese, Italy) plethysmometer was used to determine the paw volume (mL) of both the ipsilateral and contralateral hind paws. Rats were lightly restrained, and the contralateral paw was measured first followed by the ipsilateral paw. Data are expressed as the difference between these two measurements.

### Microvessel isolation

Cerebral microvessels were isolated as previously described [20]. Briefly, rats were anesthetized, decapitated, and the brains removed. Brains were minced and homogenized using a Potter-Elvjehm homogenizer. Samples were layered over 30% Ficoll and centrifuged (20 min at 5800 x g at 4°C) to remove the majority of the lipids. The vessels in the pellet were resuspended in buffer and filtered using a series of nylon mesh filters. These limited the remaining vessels to those between 300 μm and 40 μm. Samples were frozen at -20°C or used immediately for a subsequent biochemical analysis.

### Nuclear/cytosolic protein analysis

Animals of like treatment were pooled to create samples consisting of 3 independent rats coming from different cages. Nuclear and cytosolic fractions from the isolated microvessels were separated using the NE-PER Nuclear and Cytoplasmic Extraction Kit (ThermoFisher Scientific, Carlsbad, CA) according to the provided instructions.

### Western blot and quantification

Equal concentrations of nuclear and cytosolic protein, as determined by the Pierce BCA Protein Assay Kit (ThermoFisher Scientific), were loaded onto Criterion TGX 4–20% gels (Bio-Rad) and separated via SDS-PAGE gel electrophoresis. Proteins were detected and quantified using antibodies to MDR1 (sc8313) and nucleoporin p62 (sc25523) from Santa Cruz Biotechnology (Santa Cruz Biotechnology, Dallas, TX), nucleoporin p62 (ab95956) from Abcam (Abcam, Cambridge, MA), and α-tubulin (#2144) from Cell Signaling Technology (Cell Signaling Technology, Inc., Danvers, MA). An HRP-linked anti-rabbit secondary (GE Healthcare, Piscataway, NJ) was used for the detection of bound antibodies. Proteins were measured by chemiluminescence using the Clarity bioluminescence kit (Bio-Rad) and imaged on a ChemiDoc System (Bio-Rad). The bands were quantified following removal of background signal using the algorithms in FIJI [25].These images were cropped and the contrast and brightness adjusted for the entire cropped portion before constructing the figure. P-gp was normalized to nucleoporin in nuclear samples and α-tubulin in cytosolic, microvessel isolate and whole brain cortex samples as previously described [21].

### Statistics

Difference between means was tested using the Student’s t-test using the algorithms in Microsoft Excel (Microsoft, Redmond, WA). When a comparison required multiple t-tests the Dunn-Bonferroni method was used to control the Type I error [26]. Significance was set at p ≤ 0.05 unless otherwise stated. We used historical data to run through GPower 3.1 (Universität Düsseldorf, Düsseldorf, North Rhine-Westphalia, Germany) for each modality to determine minimal group size for 80% statistical power and significance, when alpha = 0.05 for a 20% difference between treatment groups [27]. For the combination of inflammation and the mechanical and thermal measurements, n = 8 rats is the minimal number required. All graphs were produced using GraphPad Prism 7 (GraphPad Software, Inc., La Jolla, CA). Raw data are contained in S1 Dataset.

## Results

### Surgical implantation of osmotic minipumps models chronic morphine exposure

To test whether p-gp trafficking was affected by chronic opioid exposure our first step was to establish a chronic morphine exposure model in female rats. We surgically implanted osmotic minipumps that delivered 5 mg/kg/day morphine for six days. Using the up-down method of von Frey mechanical sensitivity [22], we found that animals that received a morphine pump had a 50% reduction in mechanical thresholds after morphine exposure from a pre-surgery value of 13.50 g +/- 1.17 g to a post-surgery value of 6.73 g +/- 1.28 g (p < 0.001: n = 8). Animals implanted with a saline-filled minipump showed no significant decrease in mechanical thresholds following exposure (pre-surgery value = 14.88 g +/- 0.12 g and post-surgery value = 13.34 g +/- 1.10 g; n = 8). There was no difference in pre-surgery mechanical sensitivity between the two groups. These data are consistent with the expected responses to chronic morphine exposure.

### Chronic morphine exposure eliminates the acute morphine effect on mechanical allodynia

**Fig 1:**
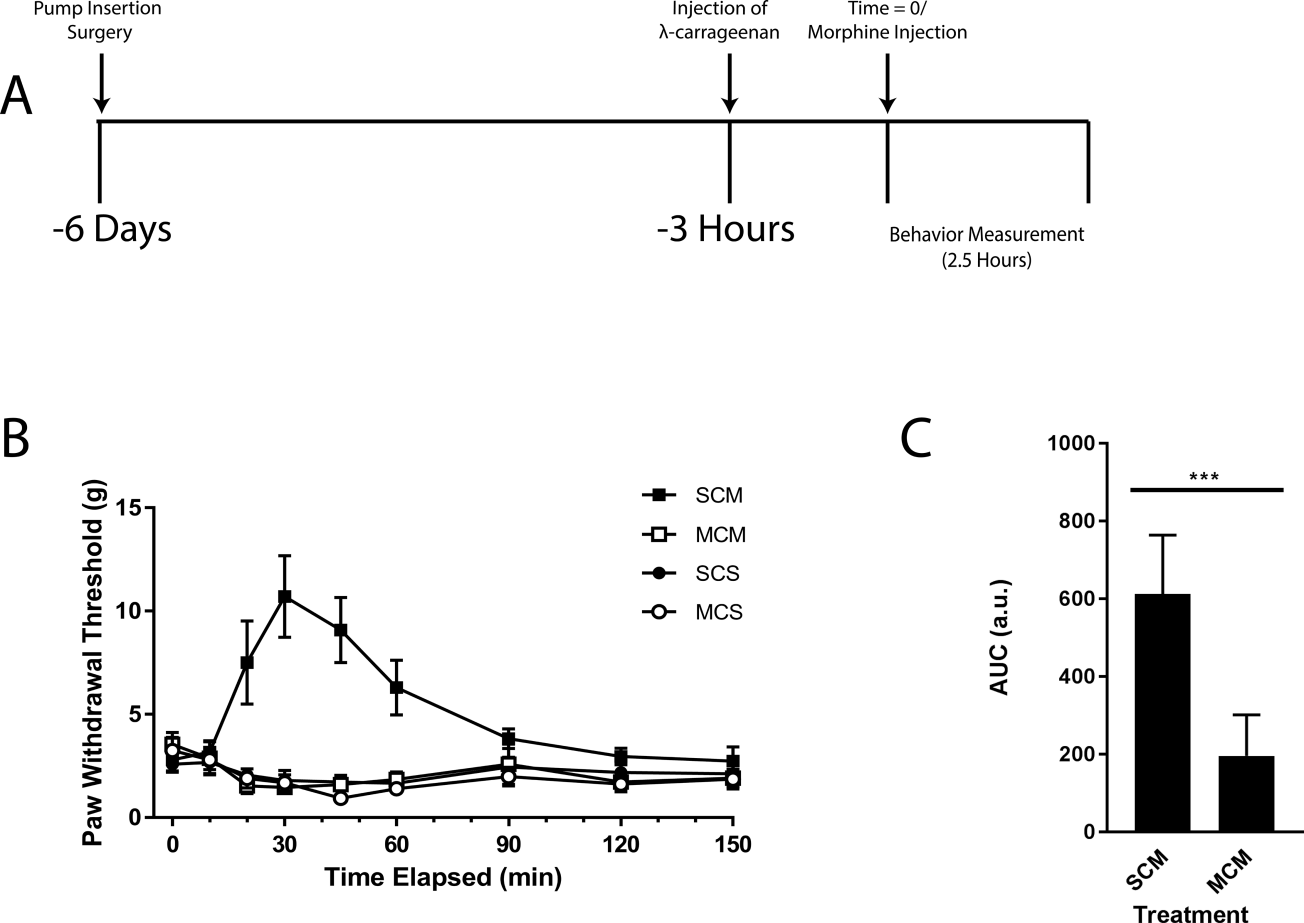
Prolonged morphine exposure eliminates the antinociceptive effect of acute morphine administration on mechanical sensitivity. **(A)** Timeline of treatments used in these experiments. **(B)** Mechanical ipsilateral paw withdrawal threshold determined by the von Frey mechanical sensitivity test in rats exposed to 5 mg/kg/day of morphine or saline for 6 days, then treated with an acute dose (2.5 mg/kg) of morphine or saline (time = 0) 3 hours after injection of λ-carrageenan into the left hind paw. The symbols mean: SCS: Saline osmotic minipump (24 μL/day)/ λ-carrageenan hind paw injection (0.1 mL)/ Saline intraperitoneal injection (1 mL/kg);SCM: Saline osmotic minipump (24 μL/day)/ λ-carrageenan hind paw injection (0.1 mL)/ Morphine intraperitoneal injection (1mL/kg) (2.5 mg/kg);MCS: Morphine osmotic minipump (24 μL/day) (5mg/kg/day)/ λ-carrageenan hind paw injection (0.1 mL)/ Saline intraperitoneal injection (1 mL/kg);MCM: Morphine osmotic minipump (24 μL/day) (5mg/kg/day)/ λ-carrageenan hind paw injection (0.1 mL)/ Morphine intraperitoneal injection (1 mL/kg) (2.5 mg/kg). Values are the mean +/- SEM (n = 8). **(C)** The area under the curve for the animals treated with SCM and MCM during the peak observed morphine effect (between 20 minutes and 60 minutes). Values are the mean + SEM (n = 8). *** denotes significantly different (p<0.0001).

If the animals are tolerant to morphine we would predict that an acute dose of morphine would not alleviate the pain response caused by PIP. To test this prediction, we injected λ-carrageenan into the left hind paw, waited 3h for the reaction to develop and measured the ability of an acute dose of morphine (2.5 mg/kg, I.P.) to suppress the increase in hind paw mechanical allodynia (Fig 1A). Control animals that received a minipump containing saline, a saline paw injection and a saline injection I.P. did not develop any mechanical allodynia across the full experimental time course (S1 Fig). These animals showed a paw withdrawal threshold average of 15 g throughout the timecourse. For the animals that received a λ-carrageenan injection, the mechanical allodynia values 3h post-injection were enhanced (paw withdrawal threshold of approximately 3 g) compared to those that received a saline paw injection (Fig 1B: time = 0). All the λ-carrageenan injected animals had similar mechanical sensitivity independent of the contents of the subscapular minipump. Animals that received a pump containing saline, a λ-carrageenan paw injection and a saline I.P. injection showed greater mechanical allodynia over the entire timecourse of the behavior measurements (Fig 1B: SCS). Carrageenan-induced mechanical allodynia was attenuated by an acute dose of morphine in animals after saline pre-exposure (Fig 1B: SCM). The onset of the antinociceptive effect of the acute morphine dose in animals with a saline-containing minipump was 20 min post injection and lasted for 40 minutes.

Rats pre-exposed to morphine then given the acute dose after λ-carrageenan were indistinguishable from those that did not receive acute morphine (Fig 1B). A comparison of the area under the curve for these animals to that in animals that received a saline-containing minipump prior to λ-carrageenan and acute morphine injections showed a significantly larger area for the morphine naïve animals (p < 0.0001; Fig 1C). Animals with no injection of λ-carrageenan tended to have a lower mechanical threshold when exposed to morphine for six days than those exposed to saline; however, this difference was not statistically significant (S1 Fig). These data indicate that sustained exposure to a moderate dose of morphine (5 mg/kg/day) for 6 days prior to λ-carrageenan abolishes the antinociceptive effects of acute morphine on mechanical allodynia.

### Chronic morphine exposure eliminates the acute morphine effect on thermal sensitivity

**Fig 2:**
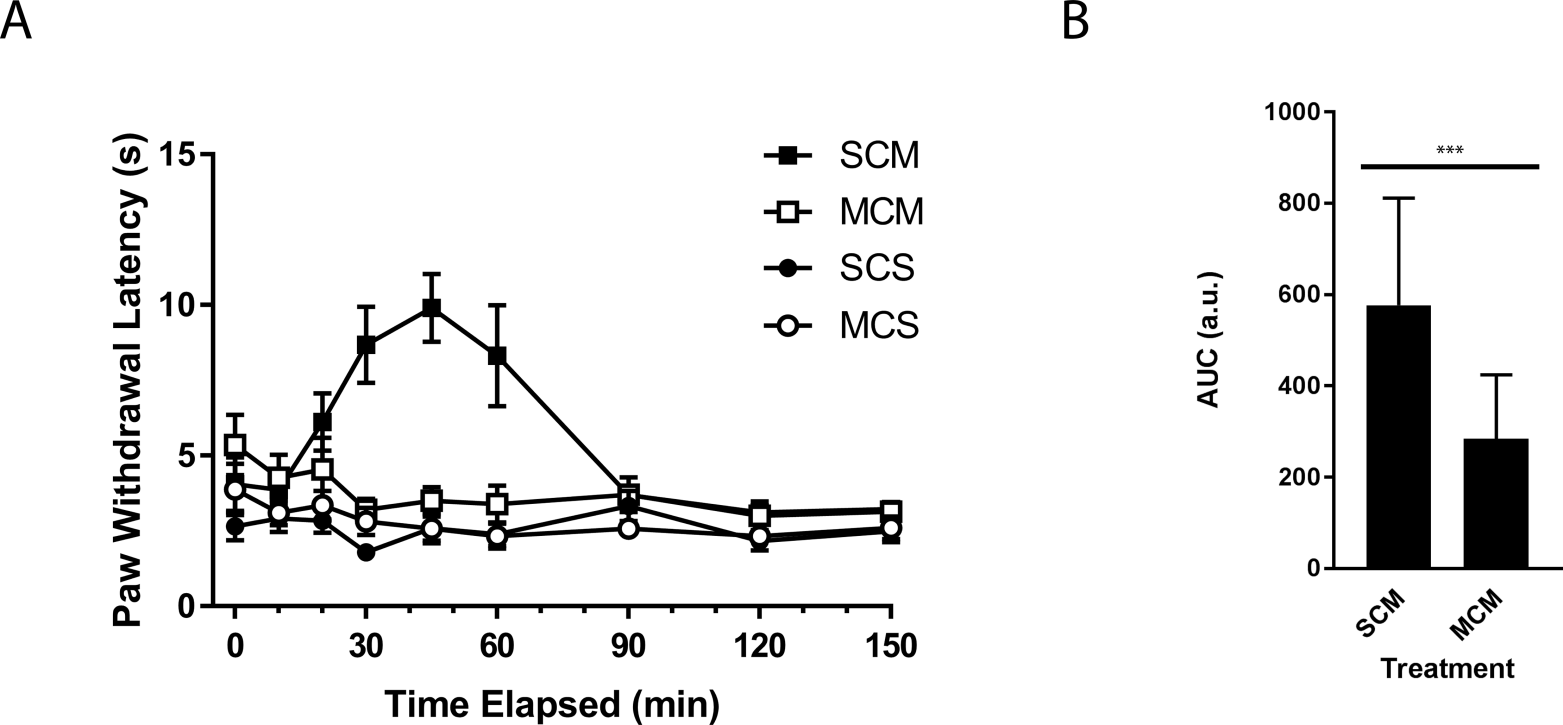
Prolonged morphine exposure eliminates the antinociceptive effect of acute morphine administration on thermal sensitivity. **(A)** Thermal paw withdrawal threshold was determined by the Hargraves thermal sensitivity test in the ipsilateral paw of rats exposed to 5 mg/kg/day of morphine or saline for 6 days, then treated with an acute dose (2.5 mg/kg) of morphine or saline (time = 0) 3 hours after injection of λ-carrageenan into the left hind paw. The symbols mean: SCS: Saline osmotic minipump (24 μL/day)/ λ-carrageenan hind paw injection (0.1 mL)/ Saline intraperitoneal injection (1 mL/kg);SCM: Saline osmotic minipump (24 μL/day)/ λ-carrageenan hind paw injection (0.1 mL)/ Morphine intraperitoneal injection (1 mL/kg) (2.5 mg/kg);MCS: Morphine osmotic minipump (24 μL/day) (5 mg/kg/day)/ λ-carrageenan hind paw injection (0.1 mL)/ Saline intraperitoneal injection (1 mL/kg);MCM: Morphine osmotic minipump (24 μL/day) (5 mg/kg/day)/ λ-carrageenan hind paw injection (0.1 mL)/ Morphine intraperitoneal injection (1 mL/kg) (2.5 mg/kg). Values are the mean +/- SEM (n = 9). **(B)** The area under the curve for the animals treated with SCM and MCM during the peak observed morphine effect (between 30 minutes and 60 minutes). Values are mean + SEM (n = 9). *** denotes significantly different (p = 0.007).

The inability of an acute dose of morphine to reduce thermal sensitivity in animals previously exposed to morphine is also a characteristic of this model in male rats [28]. Using the same time course as the mechanical sensitivity experiment, we determined thermal sensitivity with the Hargreaves method [24]. Animals exposed to morphine before λ-carrageenan injection showed no change in thermal sensitivity between pre- and post-surgery measurements (11.49 s +/- 2.76 s and 10.4 s +/- 2.12 s, respectively). Animals that received a saline pump similarly showed no difference between pre-and post-surgery values (10.06 s +/- 1.69 s to 10.71 s +/- 1.62 s, respectively). Animals only exposed to saline averaged a paw withdrawal latency of approximately 10 s for the ipsilateral paw throughout the time course (S2 Fig). Rats that had been pre-exposed to morphine prior to λ-carrageenan injection had a greatly reduced antinociceptive response to morphine compared to morphine naïve animals (Fig 2A: MCM). Animals that did not receive an acute dose of morphine did not have a change in sensitivity in the ipsilateral paw (SCS, MCS). For thermal sensitivity, an antinociceptive effect was seen from 30 to 60 minutes in the animals with pre-exposure to saline (SCM). The area under the curve was larger for the animals with no pre-exposure to morphine than for those that were exposed to morphine prior to λ-carrageenan paw injections and an acute morphine dose (p < 0.01) (Fig 2B). Animals with no injection of λ-carrageenan showed no change over the time course (S2 Fig). These data further support loss of acute antinociceptive effects of morphine against thermal stimuli after sustained use.

### Chronic morphine exposure does not affect paw edema in a λ-carrageenan induced model of peripheral inflammatory pain

**Fig 3:**
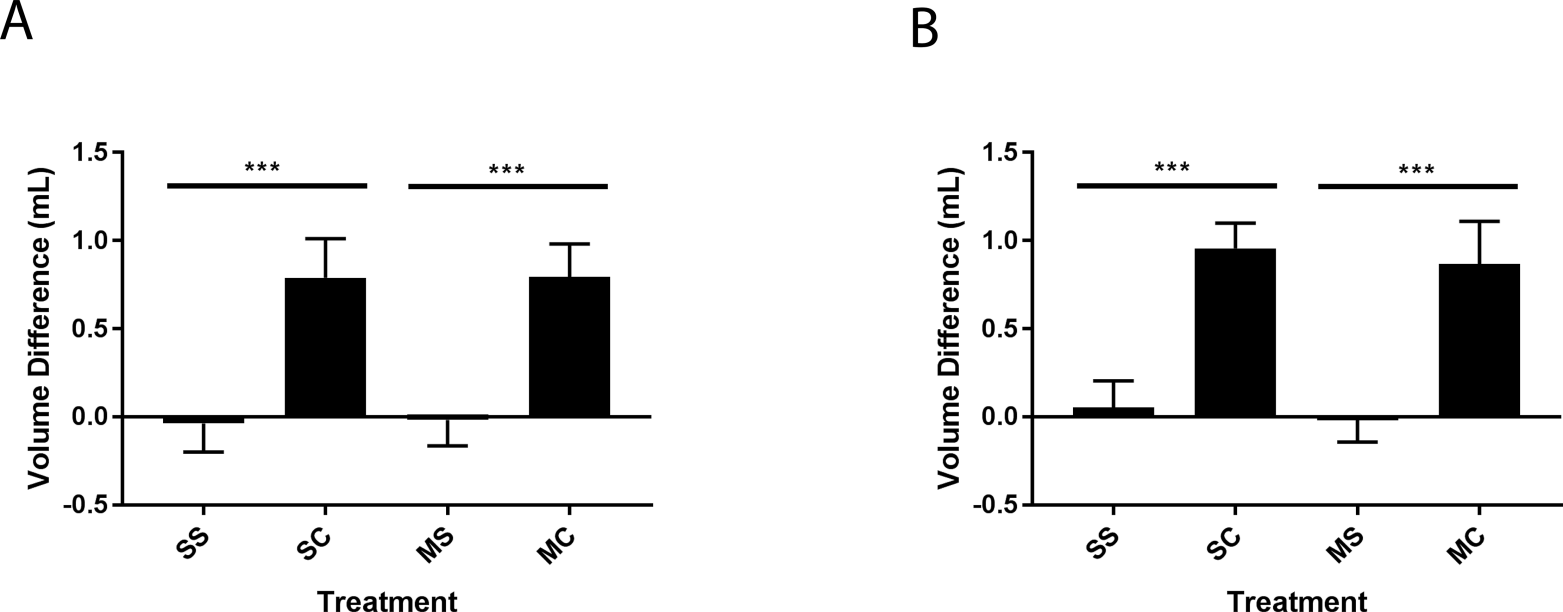
Chronic morphine exposure has no effect on hind paw edema following λ-carrageenan injection. **(A)** Animals tested for mechanical sensitivity. **(B)** Animals tested for thermal sensitivity. The symbols mean: SS: Saline osmotic minipump/ Saline hind paw injection (100 μL); SC: Saline osmotic minipump/ λ-carrageenan hind paw injection (100 μL); MS: Morphine (5 mg/kg/day) osmotic minipump/ Saline hind paw injection (100 μL); MC: Morphine (5 mg/kg/day) osmotic minipump/ λ-carrageenan hind paw injection (100 μL). Values are the mean + SEM (A: n = 16, B: n = 18). *** denotes significantly different (p<0.0001).

While previous studies have shown that the pain component of the PIP stimulus is the important component for the effect on the BBB, the inflammation and edema may play an important role in the magnitude of the pain. To ensure long-term morphine exposure did not affect the swelling in the paw, the volume of both feet was measured as a way to determine paw edema. At 3 hours post injection of λ-carrageenan, the ipsilateral paw volume, as measured by plethysmometry, was increased by 0.79 +/- 0.06 and 0.80 +/- 0.05 mL relative to the contralateral paw in the saline exposed and morphine-exposed animals used for the mechanical sensitivity experiment, respectively (Fig 3A). At 3 hours post injection of λ-carrageenan, the ipsilateral paw was increased by 0.96 +/- 0.03 and 0.87 +/- 0.06 mL relative to the contralateral paw in the saline exposed and morphine-exposed animals used for the thermal sensitivity experiment, respectively (Fig 3B). Animals that received a paw injection of saline did not have a significant difference in paw volume, regardless of treatment, demonstrating that chronic morphine exposure has no effect on paw edema.

### Chronic morphine exposure does not change p-gp expression in whole brain cortex samples or in microvessel isolate samples

**Fig 4:**
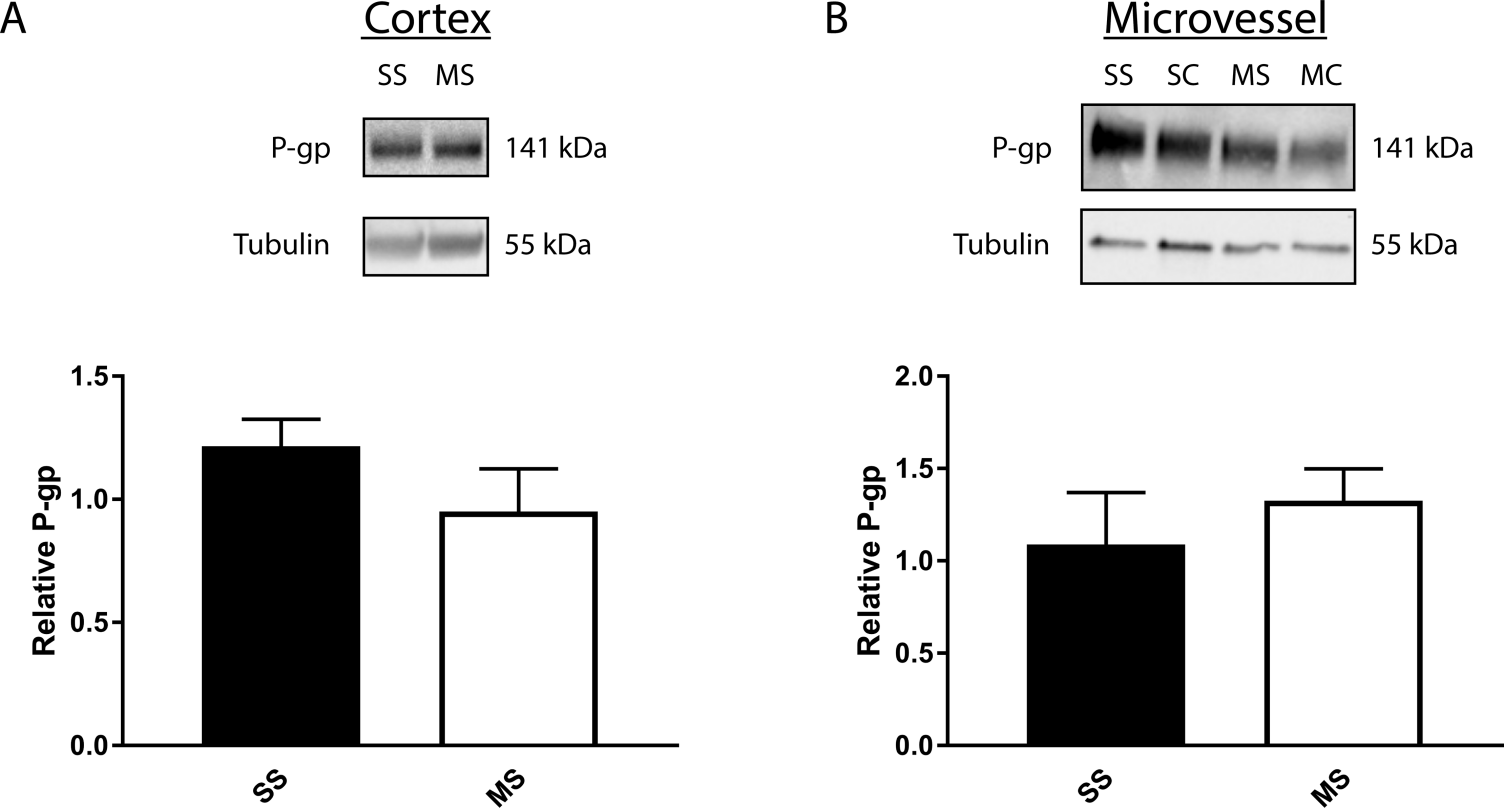
Chronic morphine exposure has no effect on total p-gp expression in the whole brain or microvessel isolates. **(A)** Representative immunoblot indicating p-gp and tubulin as a loading control in whole brain cortex. P-gp expresion normalized to tubulin in whole brain cortex samples. Values are the mean + SEM (n = 3 pools of 3 rats each) **(B)** Representative immune blot indicating p-gp and tubulin in microvessel isolates. P-gp expresion normalized to tubulin in microvessel isolate samples. Values are the mean + SEM (n = 3 pools of 3 rats each).

Long-term opioid exposure increases p-gp expression in brains of male rats [11,14]. To determine whether an increase in p-gp amount could contribute to the loss of acute antinociceptive effects of morphine we observed in our female rats, we measured p-gp in whole brain cortex and microvessel isolates in the absence of PIP to assess the effect of sustained morphine on p-gp expression. A comparison of whole brain cortex isolates from animals that received either saline- or morphine-containing minipumps followed by a saline paw injection showed no significant change in total p-gp (Fig 4A) (p = 0.758). The expression of p-gp in isolated microvessels was also unchanged by treatment (Fig 4B) (p = 0.457).

### PIP mediated trafficking of p-gp from the nucleus is increased by long term opioid exposure

**Fig 5:**
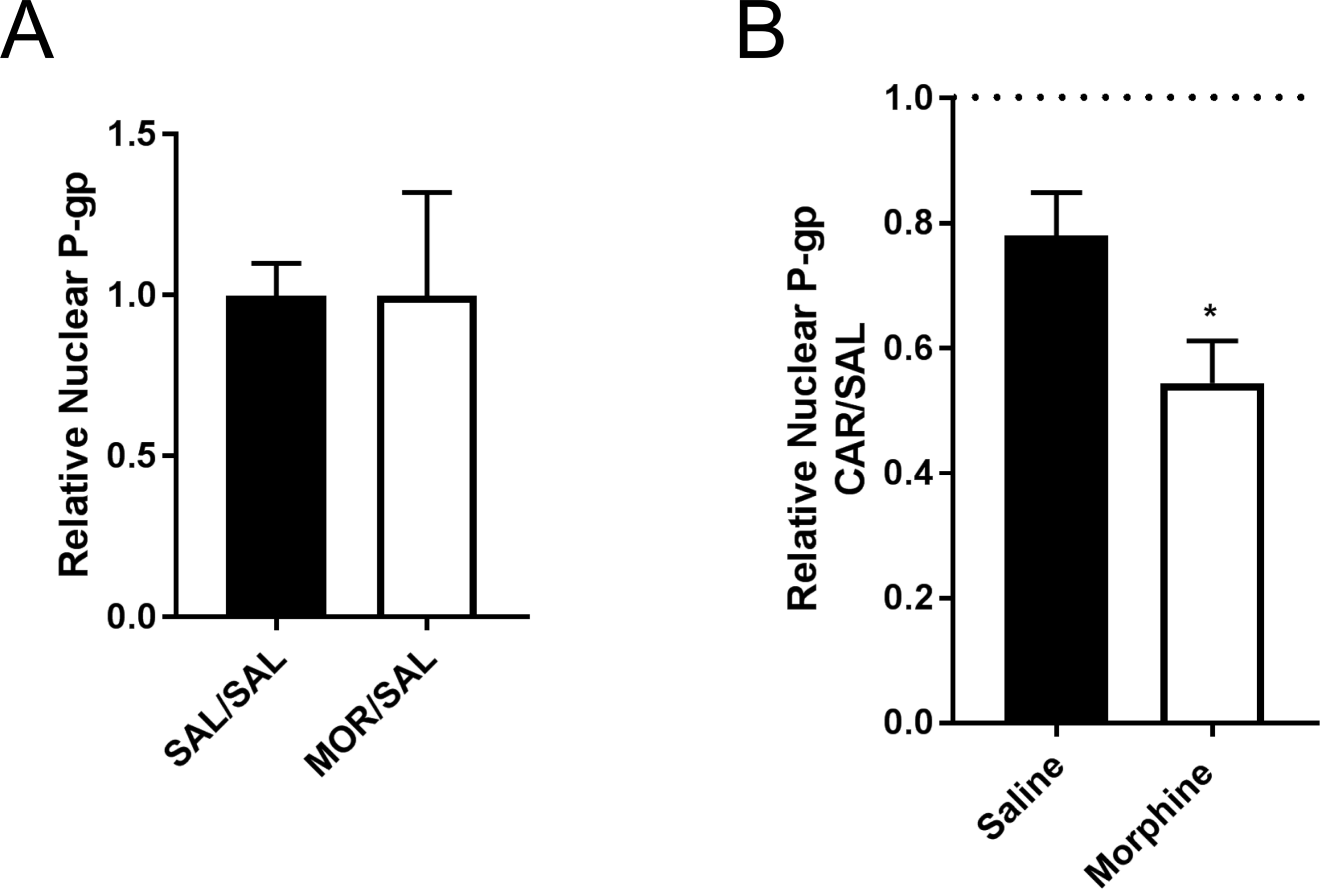
Chronic morphine exposure increases trafficking of p-gp away from the nucleus after peripheral inflammatory pain. **(A)** P-gp expresion normalized to nucleoporin in the nuclear fractions. Values are the mean + SEM (n = 3 pools of 3 rats each) **(B)** Ratio of nuclear p-gp normalized to nulcoporin in CAR/SAL injected animals as a measure of p-gp trafficking. The dashed line indicates the value if the nuclear p-gp in saline and ʎ-carrageenan injected animals was equal. Values are the mean + SEM (n = 3 pools of 3 rats each). *** denotes significantly different from control (saline) (p<0.05). The symbols mean: SAL/SAL represents animals with an osmotic minipump filled with 0.9% saline and a 0.9% saline hind paw injection. MOR/SAL represents animals with an osmotic minipump filled with morphine (5 mg/kg/day) in 0.9% saline and a 0.9% saline hind paw injection.

PIP is sufficient to induce trafficking of p-gp away from nuclear reservoirs [21]. We tested whether sustained morphine exposure altered this trafficking. Using a nuclear protein isolation assay, we measured nuclear p-gp after chronic morphine treatment. As shown by the nucleoporin, a nuclear marker, and α-tubulin, a cytosolic protein, we were able to separate the two fractions from cells in the microvessel isolate (S3 Fig). Quantitation of nuclear p-gp showed that a six-day morphine exposure did not change the nuclear p-gp (Fig 5A). Animals exposed to morphine showed a 46% decrease in nuclear p-gp when given a PIP stimulus (Fig 5B). Animals with a saline pump showed a 24% reduction in nuclear p-gp when exposed to PIP (Fig 5B). These data indicate increased trafficking of p-gp from the nucleus in animals exposed to morphine prior to PIP.

## Discussion

**Fig 6:**
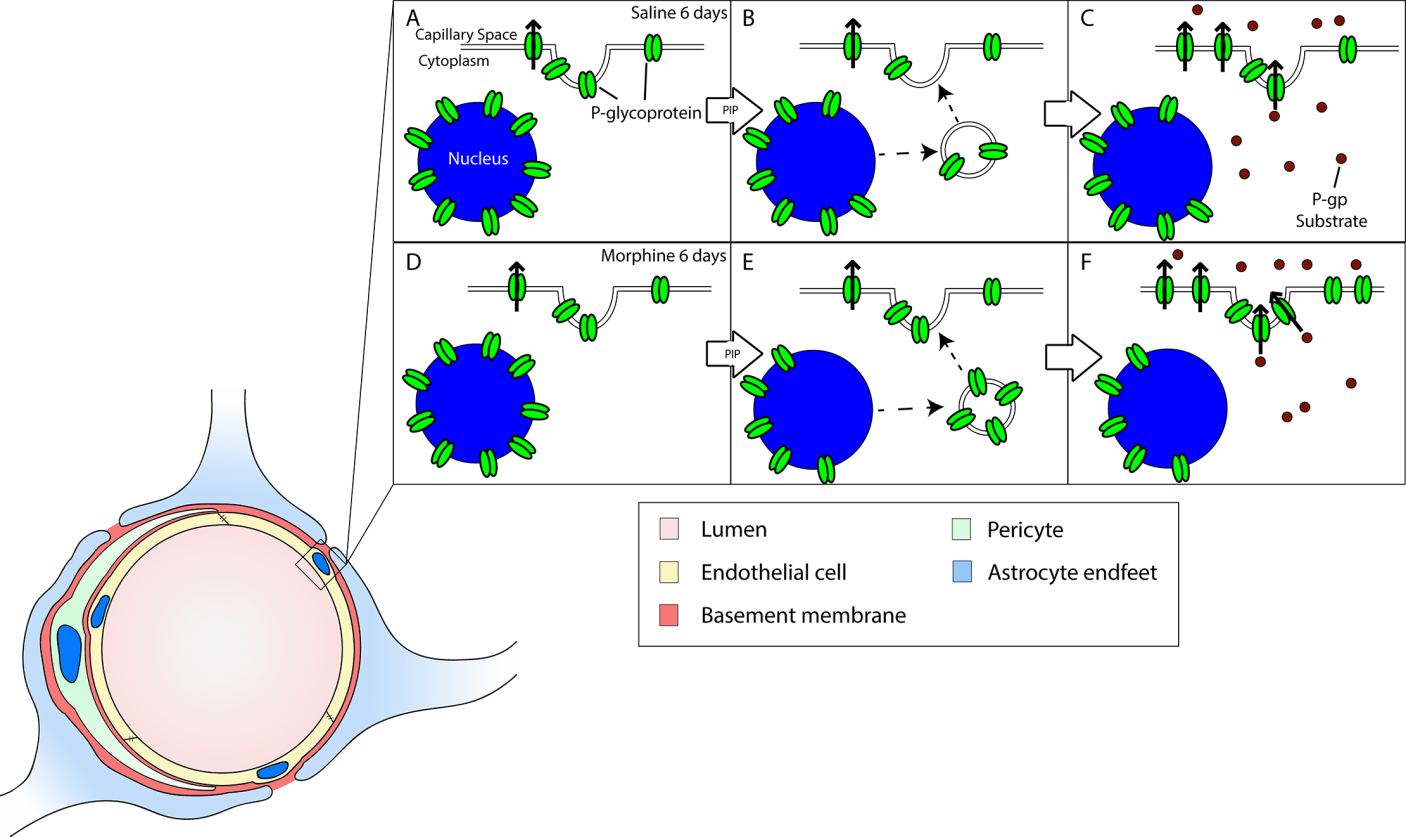
Suggested model demonstrating p-gp trafficking in the presence of peripheral inflammatory pain. In the presense of saline **(A)**, PIP induces trafficking of p-gp away from the nucleus **(B)** to the endothelial cell membrane where p-gp can efflux substrate through the membrane **(C)**. In the presense of morhine **(D)**, this trafficking is increased **(E)** resulting in a greater presense of p-gp at the cell membrane and an increase in substrate efflux **(F)**.

Long-term opioid exposure is sufficient to induce a 2-fold increase in PIP-mediated trafficking of p-gp away from the nucleus in rat brain microvessel isolates. PIP induces p-gp trafficking from nuclear reservoirs to the plasma membrane [21] concomitant with increased p-gp activity and decreased morphine analgesic efficacy [12]. In morphine tolerant rats, an acute dose of morphine is insufficient to suppress the mechanical and thermal sensitivity caused by the PIP stimulus. Increased trafficking of p-gp from nuclear reservoirs could contribute to increased drug efflux that reduces morphine analgesic efficacy. Fig 6 depicts the predicted consequences of increased p-gp trafficking due to long-term morphine exposure. These data suggest a potential role of p-gp trafficking in the clinical challenges associated with decreased opioid analgesic efficacy in patients with a history of long-term opioid use when there is a need for acute pain management [29,30].

Our model, 6 days exposure to morphine (5mg/kg/day delivered at a rate of ~ 0.208 mg/h) from osmotic minipumps [31], was sufficient to induce morphine tolerance in female rats. Using measurements of mechanical allodynia and thermal sensitivity, we determined that the effective window for an acute dose of morphine was from 20 to 60 minutes post-injection in naïve animals. Animals that had been pre-exposed to morphine and received an acute dose of morphine were indistinguishable from those that received saline. The elimination of the antinociceptive effect of morphine at this dose is a clear demonstration of morphine tolerance.

Chronic opioid exposure is characterized in part by decreased uptake of opioids into the CNS. There is considerable evidence that there is an increase in p-gp expression in the brain of both rats and mice with long-term exposure to opioids [11,14,32]. These changes are region specific and mostly occur in regions of the cortex, hippocampus and large vessels of the brain. Although we found no change in p-gp in the whole brain cortex sample, we did not investigate the specific regions where others have seen an increase [14,15]. Similar to our observations in this study, other studies did not find an increase in total p-gp expression in isolated microvessels in opioid tolerant animals [14,15]. These data indicate the alterations in p-gp trafficking we observe are independent of protein amount.

The 2-fold increase in PIP-mediated p-gp trafficking caused by long-term morphine exposure is expected to further lower morphine analgesic efficacy in long-term opioid-treated patients experiencing acute pain. PIP decreases opioid analgesic efficacy and causes trafficking of p-gp from the nucleus to the luminal surface of BBB endothelial cells in non-opioid tolerant animals concomitant with decreased CNS opioid uptake [12,21]. An increase in the p-gp trafficking to the capillary luminal surface in opioid-tolerant rats positions additional p-gp protein molecules in a location to augment efflux of opioids into the blood. In the luminal membrane, p-gp binds to drug from inside the endothelial cells and as it is diffusing through the endothelial cell membrane from the blood [33]. Then, p-gp effluxes drug back into the circulation [33]. There are ~650 km of cortical microvessels [20]. A 46% increase in luminal p-gp would result in a substantial increase in drug efflux potential when multiplied over the surface area of the cortical microvessels. A potentiated efflux would further decrease morphine efficacy in opioid tolerant rats experiencing an acute pain stimulus. Because morphine is a p-gp substrate, these observations suggest a relationship between inflammatory pain induced p-gp trafficking and the decrease in morphine efficacy in animals in pain. An increase in acute p-gp trafficking following long-term exposure to morphine would explain the further decrease in the efficacy of opioid analgesics. Less morphine can reach the CNS targets needed for analgesia in animals that have attenuated analgesic signaling due to morphine pre-exposure.

Pain management is an essential component of recovery from surgery or injury. The observation that chronic exposure to morphine increases PIP induced p-gp trafficking needs to be taken into account when treating acute pain in patients with long-term opioid use. A decreased ability of opioids to reach their target due to acute pain signaling would require dose escalation to elicit the same analgesic effect in these patients. Our data also has application to post-surgical pain management in non-opioid exposed patients. In animals not pre-exposed to morphine we see increased p-gp trafficking to the lumen ([21] and the current study), although this is to a lesser degree than in the opioid-exposed animals. Decreased ability of opioids to reach the CNS analgesic targets for any type of pain management could contribute to dose escalation, misuse and abuse. With opioid use and the number of opioid prescriptions at all-time highs, there is an ever growing need for strategies to improve pain management in opioid tolerant patient populations [34,35] and opioid naïve populations so that tolerance does not occur.

Changes to p-gp trafficking induced by long-term exposure to morphine may have ramifications for other therapeutic combinations. P-gp has a large number of substrates and this may be applicable to many of them [36]. Although the focus of the current study was pain management and opioids, CNS delivery of other therapeutics that are p-gp substrates would be similarly affected in opioid tolerant patients experiencing acute pain. Chronic administration of Rifampicin, an antibiotic that is also a p-gp substrate, increases whole brain p-gp expression in a way similar to morphine [32]. This suggests the mechanism may be triggered by the presence of a substrate, not due to a unique effect of a given compound. Characterization of the mechanism underlying long-term exposure to a p-gp substrate and increased p-gp trafficking induced by pain could have additional clinical implications.

One potential acute pain management strategy in opioid tolerant patients is to administer a dose of a p-gp inhibitor at the time of opioid administration. Several compounds that utilize different mechanisms of p-gp inhibition including blocking drug binding sites, changing cell membrane lipids or inhibiting the hydrolysis of ATP have been developed [37]. Inhibiting p-gp overcomes multi-drug resistances in cancer cells associated with p-gp [38–40]. Animal studies suggest p-gp inhibition has potential in pain management. Mice lacking p-gp have both increased and prolonged opioid-induced analgesia compared to wild-type mice [41]. Several studies investigating the effect of a variety of p-gp inhibitors [42,43] indicate p-gp inhibition results in increased CNS morphine uptake combined with increased antinociceptive effect. However, one of the major caveats is that agents that directly inhibit p-gp have not proven clinically viable in the past because patients die of xenobiotic toxicity and infection if basal p-gp activity is not maintained [44,45]. For these agents to be effective and safe for acute pain management in opioid tolerant patients, care is required in selecting the appropriate dose and patient monitoring is paramount. Other strategies that block an increase in p-gp at the transcriptional, translational and post-translational level also have clinical potential [10,46] and may circumvent the toxicity issues inherent in direct p-gp inhibition.

The increased trafficking of p-gp suggests a novel target for therapeutic intervention to improve pain management while reducing opioid dose escalation. Although the trafficking signals and mechanisms are not yet characterized, our data suggest that identifying potential drug targets in this process could result in a clinical application. Attenuation of p-gp translocation from the nucleus would increase the delivery of drugs into the brain by preventing increased efflux. This method of p-gp modulation would spare the basal levels of p-gp. Preserving the basal level of p-gp ensures the protective nature of this protein is intact. Blocking the trafficking would mitigate the detrimental effect p-gp has on drug delivery to improve acute opioid analgesic efficacy. Acute management of p-gp activity would be a valuable clinical tool for pain treatment not only in patients with chronic opioid use, but also in others undergoing chronic intervention with other p-gp substrates and help combat the opioid epidemic.

## Supporting information

S1 Fig Chronic morphine administration tends to reduces mechanical sensitivity thresholds in the ipsilateral paw. The symbols mean: SSS: Saline osmotic minipump (24 μL/day)/ saline hind paw injection (0.1 mL)/ Saline intraperitoneal injection (1 mL/kg); SSM: Saline osmotic minipump (24 μL/day)/ saline hind paw injection (0.1 mL)/ Morphine intraperitoneal injection (1 mL/kg) (2.5 mg/kg);MSS: Morphine osmotic mini-pump (24 μL/day) (5 mg/kg/day)/ saline hind paw injection (0.1 mL)/ Saline intraperitoneal injection (1 mL/kg);MSM: Morphine osmotic minipump (24 μL/day) (5 mg/kg/day)/ saline hind paw injection (0.1 mL)/ Morphine intraperitoneal injection (1 mL/kg) (2.5 mg/kg). Values are mean +/- SEM (n = 8).
(TIF)

S2 Fig Chronic morphine administration had no effect on thermal paw withdrawal measurements in the ipsilateral paw. The symbols mean: SSS: Saline osmotic mini-pump (24 μL/day)/ saline hind paw injection (0.1 mL)/ Saline intraperitoneal injection (1 mL/kg); SSM: Saline Osmotic mini-pump (24 μL/day)/ saline hind paw injection (0.1 mL)/ Morphine intraperitoneal injection (1 mL/kg) (2.5 mg/kg);MSS: Morphine Osmotic mini-pump (24 μL/day) (5 mg/kg/day)/ saline hind paw injection (0.1 mL)/ Saline intraperitoneal injection (1 mL/kg);MSM: Morphine Osmotic mini-pump (24 μL/day) (5 mg/kg/day)/ saline hind paw injection (0.1 mL)/ Morphine intraperitoneal injection (1 mL/kg) (2.5 mg/kg). Values are the mean +/- SEM (n = 9).
(TIF)

S3 Fig Representative immunoblot indicating the purity of the nuclear fractions from microvessel isolates. Blot indicates the relative amount of, nucleoporin, a nuclear membrane marker, and tubulin, a cytosolic protein, in nuclear (N) and cytosolic (C) fractions from a microvessel isolate also probed for p-glycoprotein.
(TIF)

S1 Dataset Raw data files that are the basis of each figure. Files are labeled by figure number.
(PDF)
