# Supplementary figures and images for: Chronic morphine exposure potentiates p-glycoprotein trafficking from nuclear reservoirs in cortical rat brain microvessels
Source: PLoS One. 2018 Feb 7;13(2):e0192340. doi: 10.1371/journal.pone.0192340 (PMC5802945; doi:10.1371/journal.pone.0192340)

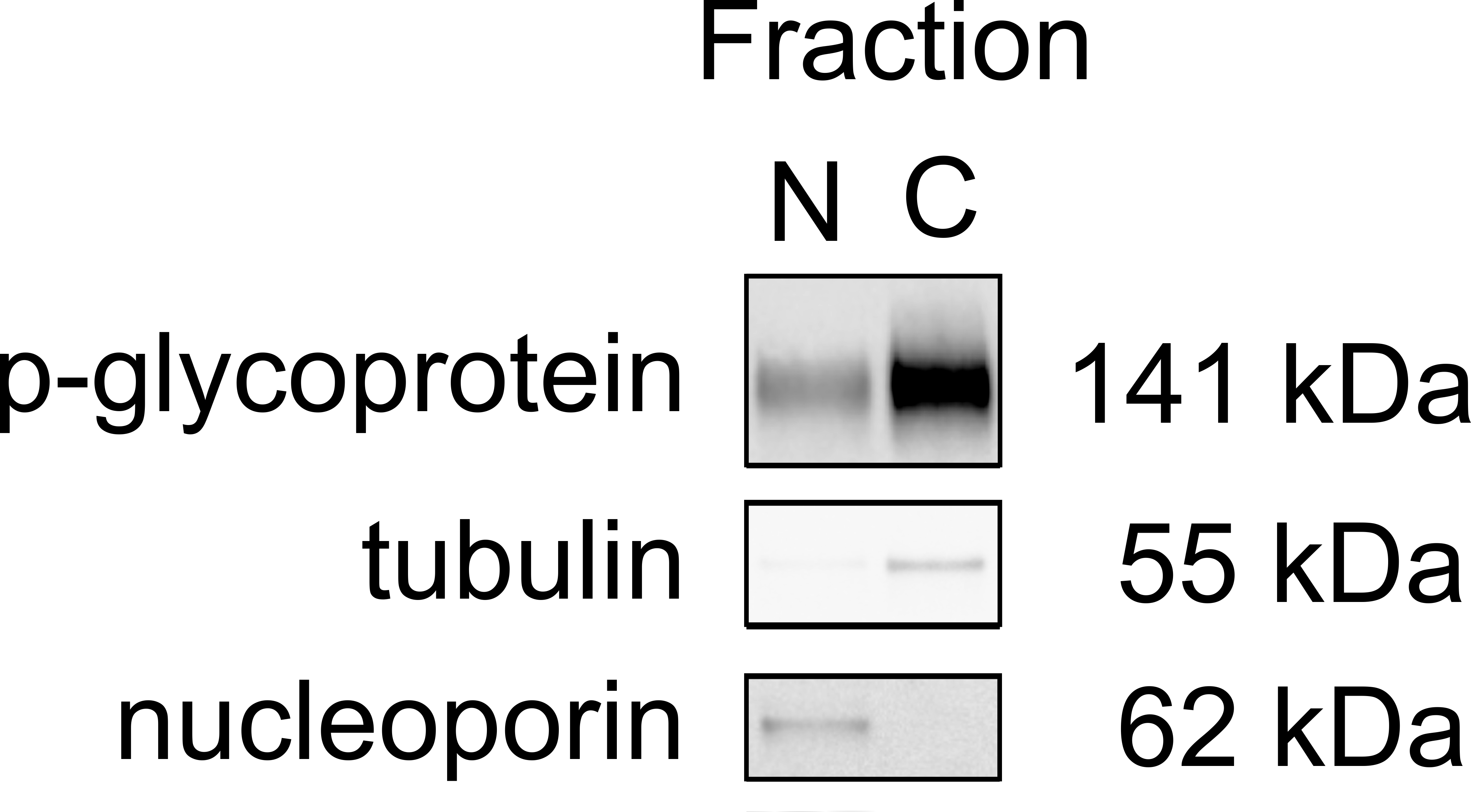

Supplement: S3 Fig — Blot indicates the relative amount of, nucleoporin, a nuclear membrane marker, and tubulin, a cytosolic protein, in nuclear (N) and cytosolic (C) fractions from a microvessel isolate also probed for p-glycoprotein. (TIF) [file pone.0192340.s003.tif]
