# Supplementary material for: Chronic morphine exposure potentiates p-glycoprotein trafficking from nuclear reservoirs in cortical rat brain microvessels
Source: PLoS One. 2018 Feb 7;13(2):e0192340. doi: 10.1371/journal.pone.0192340 (PMC5802945; doi:10.1371/journal.pone.0192340)
Supplement: S1 Dataset — Files are labeled by figure number. (PDF) [file pone.0192340.s004.pdf]

Supplementary data 4 (Raw data for figures)

Fig 1

	Rat 1 (SAL/SAL/SAL)	Rat 2 (SAL/CAR/SAL)	Rat 3 (SAL/SAL/MOR)	Rat 4 (SAL/CAR/MOR)	Rat 5 (MOR/SAL/SAL)	Rat 6 (MOR/CAR/SAL)	Rat 7 (MOR/SAL/MOR)	Rat 8 (MOR/CAR/MOR)
Pre-surgery	15	13.960625	15	15	14.211125	13.24275	12.6465	14.62275
Baseline	15	13.041125	11.87325	13.03175	13.2345	13.0445	10.768625	7.653875
0	15	2.575625	14.8725	2.7985	12.28075	3.24075	10.783375	3.544875
10	14.211125	2.674125	13.015625	3.172625	10.30575	2.788625	10.2335	2.86375
20	15	2.0575	13.448375	7.503	9.559875	1.89375	10.191875	1.53575
30	15	1.796	12.4365	10.70025	10.657625	1.678	10.13975	1.451
45	15	1.717125	11.518375	9.085625	13.266125	0.92975	8.942875	1.59225
60	15	1.652125	15	6.289	11.44775	1.390375	6.938125	1.847
90	14.107625	2.445125	11.970125	3.81525	9.632	1.977375	10.103125	2.58125
120	13.7585	2.185875	13.261	2.950625	8.9055	1.622875	8.33225	1.7185
150	15	2.118625	13.47925	2.726375	14.11375	1.858625	11.337	1.901625

	SCM	MCM
	526.6775	237.545
	715.01	172.2075
	436.83	164.79
	528.8325	55.995
	458.3025	408.115
	750.45	233.6825
	863.325	103.0875
	622.8525	191.6125
Avg:	612.785	195.8794
SEM	53.71233	37.30476

Fig 2

	Rat 1 (SAL/SAL/SAL)	Rat 2 (SAL/CAR/SAL)	Rat 3 (SAL/SAL/MOR)	Rat 4 (SAL/CAR/MOR)	Rat 5 (MOR/SAL/SAL)	Rat 6 (MOR/CAR/SAL)	Rat 7 (MOR/SAL/MOR)	Rat 8 (MOR/CAR/MOR)
	Left	Left	Left	Left	Left	Left	Left	Left
Pre Surg	12.8111111	10.35555556	10.73333	10.0555556	9.622222222	10.0625	9.4375	11.48888889
Pre Injct	10.0222222	9.844444444	10.51111	10.7111111	9.677777778	11.05	9.4625	10.48888889
0 min	11.9	2.655555556	12.31111	4.05555556	10.02222222	3.875	11.2125	5.355555556
10 min	10.8555556	2.922222222	9.7	3.86666667	8.766666667	3.1125	11.15	4.266666667
20 min	9.98888889	2.844444444	10	6.12222222	9.522222222	3.35	11.175	4.544444444
30 min	9.85555556	1.788888889	10.91111	8.67777778	8.633333333	2.8125	9.775	3.211111111
45 min	10.4777778	2.588888889	11.05556	9.91111111	8.711111111	2.575	10.2125	3.5
60 min	10.3777778	2.388888889	12.08889	8.32222222	8	2.325	12.85	3.388888889
90 min	8.43333333	3.333333333	9.822222	3.68888889	8.744444444	2.5875	11.0625	3.7
120 min	8.88888889	2.166666667	8.655556	3.12222222	8.522222222	2.3375	10.1875	3.011111111
150 min	8.57777778	2.488888889	7.766667	3.23333333	8.122222222	2.6125	9.9125	3.144444444

AUC

	SCM	MCM
	268.5	108.75
	147.5	227.25
	622.5	345
	892.5	447.5
	780	532.5
	652.5	167.5
	610	160
	677.5	287.5
	540	287.5
average:	576.7778	284.8333
SEM	78.23753	46.41377

Fig 3

A

	SAL/SAL	SAL/CAR	MOR/SAL	MOR/CAR
	-0.41	0.61	-0.09	0.46
	0.1	0.42	0.09	0.85
	-0.07	0.54	-0.17	1
	-0.4	0.66	0.03	0.59
	0.1	0.99	0.07	1.21
	0.09	0.75	0.09	0.73
	0.05	1.24	0.07	1.06
	0.09	0.98	0.24	0.79
	-0.01	0.79	-0.13	0.66
	-0.12	0.45	0	0.68
	-0.09	0.7	-0.31	0.65
	-0.02	0.84	-0.17	0.77
	0.12	0.94	0.02	0.83
	-0.06	0.83	0.16	0.72
	-0.04	0.87	-0.19	0.86
	0.08	1.02	0	0.86
Average	-0.03688	0.789375	-0.01813	0.795
SEM	0.040771	0.055471	0.036529	0.046296

B

	SAL/SAL	SAL/CAR	MOR/SAL	MOR/CAR
	0.28	0.73	-0.3	0.9
	0.17	0.67	-0.02	0.99
	-0.02	1.07	0.08	0.87
	-0.01	0.74	-0.07	0.87
	0.07	0.93	0.2	0
	0.01	1.02	-0.07	0.77
	-0.13	1.08	-0.05	0.95
	-0.2	0.87	0.07	1.02
	0.16	0.94	0.07	0.96
	0.29	0.95	-0.24	1.05
	-0.17	1.04	-0.1	1.06
	-0.02	0.99	0.03	0.7
	0.03	0.78	0.14	0.76
	0.19	1.11	-0.06	0.94
	-0.14	1.1	-0.04	0.98
	0.23	1.03	0	0.8
	0.1	0.99	0.09	0.98
	0.11	1.16	-0.07	1.02
average	0.052778	0.955556	-0.01889	0.867778
sem	0.036365	0.029119	0.026986	0.063497

Fig 4

A

	ss	ms
	1.37439	1.406324
	0.931777	1.199744
	0.999847	0.342119
avg	1.102005	0.982729
sem	0.137603	0.325809

B

	1	2	3	average	sem
SS	1.37439	0.931777	1.296894	1.20102	0.136468
MS	1.406324	1.199744	1.384096	1.330054	0.06547

Fig 5

A

				avg	std err
SS	0.769862	1.308114	0.922025	1	0.160197
MS	1.619999	0.828532	0.546905	0.998479	0.321219

*****This is normalized to the average of SS*****

B

	1	2	3	avg	std err
SS vSC	0.670663	0.907693	0.708294	0.762217	0.073545
MS v MC	0.509165	0.446874	0.675288	0.543776	0.068171
